# The New Trend of State Estimation: From Model-Driven to Hybrid-Driven Methods

**DOI:** 10.3390/s21062085

**Published:** 2021-03-16

**Authors:** Xue-Bo Jin, Ruben Jonhson Robert Jeremiah, Ting-Li Su, Yu-Ting Bai, Jian-Lei Kong

**Affiliations:** 1 Artificial Intelligence College, Beijing Technology and Business University, Beijing 100048, China; jinxuebo@btbu.edu.cn (X.-B.J.); baiyuting@btbu.edu.cn (Y.-T.B.); kongjianlei@btbu.edu.cn (J.-L.K.); 2 China Light Industry Key Laboratory of Industrial Internet and Big Data, Beijing Technology and Business University, Beijing 100048, China; 3 School of Food and Health, Beijing Technology and Business University, Beijing 100048, China; rubenjohnson1988@gmail.com

**Keywords:** state estimation, model-driven, data-driven, hybrid-driven, Kalman filter, deep learning

## Abstract

State estimation is widely used in various automated systems, including IoT systems, unmanned systems, robots, etc. In traditional state estimation, measurement data are instantaneous and processed in real time. With modern systems’ development, sensors can obtain more and more signals and store them. Therefore, how to use these measurement big data to improve the performance of state estimation has become a hot research issue in this field. This paper reviews the development of state estimation and future development trends. First, we review the model-based state estimation methods, including the Kalman filter, such as the extended Kalman filter (EKF), unscented Kalman filter (UKF), cubature Kalman filter (CKF), etc. Particle filters and Gaussian mixture filters that can handle mixed Gaussian noise are discussed, too. These methods have high requirements for models, while it is not easy to obtain accurate system models in practice. The emergence of robust filters, the interacting multiple model (IMM), and adaptive filters are also mentioned here. Secondly, the current research status of data-driven state estimation methods is introduced based on network learning. Finally, the main research results for hybrid filters obtained in recent years are summarized and discussed, which combine model-based methods and data-driven methods. This paper is based on state estimation research results and provides a more detailed overview of model-driven, data-driven, and hybrid-driven approaches. The main algorithm of each method is provided so that beginners can have a clearer understanding. Additionally, it discusses the future development trends for researchers in state estimation.

## 1. Introduction

Some of the states in a system cannot be directly measured, or else, some measurements are not accurate enough due to sensor uncertainty. A sensor is a detection device that converts an actual physical quantity (the so-called state) into an electrical signal output and provides measurement data. The conversion process introduces some errors. These errors include drift error and measurement noise. The sensor’s correction can eliminate the drift error, but the measurement noise cannot be eliminated fundamentally. Thus, the output of the sensor cannot be completely consistent with the state to be measured.

State estimation is one of the most common methods for estimating the most likely value of a state based on measurements and a system. It is a prerequisite for high-level information extraction and control and has been widely used in mobile robots [1], unmanned aerial vehicles (UAVs) [2], sensor networks [3], smart grids [4], health performance detection and evaluation [5,6], etc.

State estimation methods have a long history. In 1809, Gauss proposed an optimization method called the least-squares method to determine a celestial body’s orbit from measurements [7]. Since the least-squares method does not need to know the signal’s prior statistical knowledge when estimating, the least-squares method has a wide range of applications in many fields.

In the 1940s, to control firepower, Wiener et al. [8] designed the Wiener filter in the frequency domain to achieve linear optimal dynamic estimation in a stationary random process system. Through the calculation of the Wiener–Hopf equation, the Wiener filter obtains the analytical solution of the optimal transfer function of the filter, which can suppress or gate signals containing a variety of information. However, because the Wiener filter requires that both the estimated state and the measurement conform to a stationary random process, and the Wiener–Hopf equation needs to be solved in the filtering process, the amount of calculation and the storage space required are extensive. The project is challenging to realize; therefore, it limits the applications of Wiener filters.

To overcome the shortcomings of Wiener filters, in 1960, Rudolf Emil Kalman proposed modern filtering theory [9]. He introduced state space in stochastic estimation theory, used state models to describe the relationship between states and measurements, and estimated states based on measurements using predictions and updates. Kalman filtering does not need to store all historical data. According to the state estimation at the previous moment and the current measurement information, a new estimate can be calculated according to the recursive method, which reduces the computer’s storage and calculation capacity requirements and improves real-time processing. Simultaneously, the Kalman filter can estimate one-dimensional, stationary random processes and multidimensional, nonstationary random processes [10].

The Kalman filter has been widely used because of its simplicity and ease of implementation. Because a computer will continue to accumulate and transmit rounding errors and truncation errors during the calculation process, the error covariance matrix loses its positive definiteness and results in unstable filter estimation. Therefore, researchers have successively proposed a series of numerical, robust filtering algorithms, such as square root filtering, UD (Upper-Diagonal) decomposition filtering, and singular value decomposition filtering [11]. These methods effectively improve the Kalman filter’s numerical stability while also increasing computational efficiency [12].

On the other hand, the standard Kalman filter requires an accurate model, the known statistical characteristics of the system noise. These requirements are relatively harsh in applications. In actual systems, the system cannot meet these conditions due to some uncertainties, making the Kalman filter lose its optimality, which reduces the estimation accuracy and even leads to divergence. Researchers have introduced the idea of robust control in filtering to solve this problem, thus forming a robust estimation [13].

Moreover, the standard Kalman filtering theory is only applicable to linear systems and requires that the observation equations are linear. Meanwhile, in actual engineering practice, the system is generally nonlinear. Therefore, in the 1970s, Bucy and Sunahara proposed the extended Kalman filter (EKF) [14,15,16]. The nonlinear system is linearized first and then uses the generalized Kalman filter to estimate the state. However, because the linearization process will introduce errors in the nonlinear system due to calculating the Jacobian matrix, the final state estimation accuracy will decrease. When the Jacobian matrix calculation is inaccurate, the problem of filtering divergence will also occur.

The sigma point Kalman filter (SPKF) method is a class of approximate nonlinear filtering methods based on the Gaussian distribution, including the unscented Kalman filter (UKF), central difference Kalman filter (CDKF), square-root unscented Kalman filter (SRUKF), etc. [17]. The ideas of these methods are roughly the same, of which the UKF is the most famous. It uses several sigma points for nonlinear systems to obtain the accuracy with second order.

With continuous research, the original methods have improved in the Gaussian domain filtering system. Some new methods have been proposed for approximating nonlinear characteristics. In Bayesian filtering’s general framework, the filtering process involves predictions and updates, including two integral operations. Under the Gaussian assumption, the integral is a multidimensional integral operation of the product of the equivalent Gaussian function and the system function. A new idea is provided to realize approximate Gaussian filtering: the nonlinear filtering is realized through an integral calculation. For example, Simon Haykin et al. proposed a new filtering strategy independent of the EKF and UKF, named the cubature Kalman filter (CKF) [18].

The above methods all describe the noise of a system using the Gaussian distribution. Studies have shown that the effectiveness of these methods is only valid for single-mode problems. They assume that the system state and noise are both Gaussian distributions, where one Gaussian component corresponds to one mode. However, many issues faced in the real world are often much more complicated than single-mode problems. A typical example is system noise, including process noise and observation noise, which is not a Gaussian distribution, but a gamma distribution or other complex mixed distributions [19]. Therefore, with the filtering of the above mentioned algorithms based on the Gaussian noise assumption, it is difficult to obtain a satisfactory estimation performance due to the model mismatch. In general, complex noise can be expressed as the sum of multiple Gaussian noises. Then, the EKF, UKF, and CKF are applied to obtain the Gaussian mixture (GM)-EKF, UKF, and CKF [20].

It is necessary to provide the system with the specific expression of the model and the distribution characteristics of noise for the standard Kalman filter, EKF, UKF, CKF, etc., as well as the multimode, complex, approximate Gaussian mixture filtering methods, such as GM-EKF, etc. However, when the system is complex, it is challenging to obtain these system models. Therefore, based on random sampling, another type of filter has appeared, which realizes the state’s estimation by calculating the conditional probability of the system state and realizes the conditional probability transfer through Bayes’ theorem. Its algorithm is not based on the state function’s distribution characteristics, observation function, and noise (such as the mean and variance, etc.), but the state’s conditional probability density. Its purpose is to provide the state’s probability distribution, not the functional expression of the state. This algorithm is based on Monte Carlo simulation technology and is usually called the sequential Monte Carlo method or particle filter method [21].

In this type of method, a large number of particles are generated by Monte Carlo simulation, and their distribution is used to approximate the probability distribution of the state. Its advantage lies in its strong applicability, and it can be applied to any complex system through calculating particles. Its accuracy can approach optimal estimation. It can overcome the shortcomings of traditional Gaussian filtering algorithms such as the EKF, which are more sensitive to initial value selection. By contrast, particle filters easily capture the actual state within a specific error range due to particles’ dispersion, thereby improving the filter system’s stability and convergence speed. However, the Monte Carlo simulation method is challenging to realize recursive filtering with, and the calculation amount is much more tremendous than that with the Gaussian approximation method, which affects its real-time application to some extent.

With the development of sensor technology and storage technology, especially in recent years, modern intelligent systems, such as unmanned aerial vehicles (UAVs), autonomous driving systems, etc., have been widely used. We found that there are more and more sensors in the system, and the measurement is also increasing. In the military and civil fields, many processes, such as target monitoring, detection, tracking, and identification, are based on multiple sensors’ measurements and are completed by information fusion technology [22]. Therefore, the data-driven learning network has also become an important research direction in state estimation theory.

Compared with model-based methods that require known system information, data-based methods can use big data to obtain system characteristics that cannot be described by the model. However, they abandon the established knowledge of the system and rely too much on data. When the data are insufficient or of low quality, the result is not better than that from traditional model-based estimation methods. Therefore, these two methods have their advantages and disadvantages. It is crucial to effectively combine model-driven and data-driven methods to improve the performance of state estimation effectively, i.e., use a hybrid-driven state estimation method.

Estimation methods such as the Kalman filter series, EKF, UKF, CKF, and particle filters have obtained rich published research results. They are widely used in various detection, tracking, and control systems. However, data-driven methods based on shallow networks cannot fundamentally improve the estimation performance, so related research has not been the mainstream part of research into estimation methods. Recently, the rapid development of other artificial intelligence methods was highlighted in recent years [23,24], prompting researchers to reconsider improving estimation performance by the hybrid modeling method. However, related research in this area has just begun, so there are not many related review papers.

Although there are not many, the authors are still willing to recommend the following reviews that are worth reading: Hong et al. [25] provides a survey for estimators that can produce accurate estimations for complex dynamic systems, such as airbag, debris detection, and active blast protection systems. From an application perspective, Dehghanpour et al. [26] focuses on distribution system state estimation in power systems. A few critical topics are discussed, such as mathematical problem formulation, the application of pseudomeasurements, metering instrument placement, network topology issues, the impacts of renewable penetration, and cybersecurity. It is worth emphasizing that both conventional and modern data-driven methods are reviewed in this survey. Similarly, Jin et al. [27,28] are also based on the applications. Simultaneously, the latter emphasizes the research progress in artificial intelligence in moving objects, which is more general and includes deep learning networks in unmanned autonomous mobile systems.

Unlike the above review papers, this review introduces state estimation methods without emphasizing a specific system or application background. We try to provide the development path of the state estimation method from the perspective of the method and discuss the future development trend under the premise of the current system’s universal characteristics.

This review is based on the problem of state estimation, starting with the introduction of classic estimation methods. We analyze the problems of classic estimation methods, the current research progress and problems of model-driven and data-driven methods, and discuss the state based on the combination of data-driven and model-driven methods. Finally, we put forward our ideas for future state estimation research.

The structure of this article is as follows. Section 2 and Section 3 are both for model-driven estimation methods. Section 2 describes the Kalman filter and Gaussian mixture filter, which are based on given system models. Section 3 describes the state estimation method when the system model is not accurate enough, including a robust filter and closed-loop adaptive filter. Section 4 discusses data-driven methods, including deep neural networks, the hyperparameter optimization of network models, and the ability to suppress sensor noise. Section 5 provides a detailed introduction to current hybrid drive estimation methods. Finally, in the Conclusion, we discuss the future development research direction.

## 2. State Estimation Based on a Distinct Model

The state estimation method discussed in this section is based on the unified framework of Bayesian filtering. These methods require a known model of the system: the expression of the state (the so-called process model) and the relationship between the measurements and state (the so-called measurement model). Furthermore, the distribution of the process noise and observation noise of the system must be known. When the system’s noise is Gaussian noise, the Kalman filter series can obtain satisfactory estimation results (mentioned in Section 2.1). However, if the noise becomes complex and cannot be approximated by the sum of Gaussian noise, the mixed filter is needed (mentioned in Section 2.2).

### 2.1. Kalman Filter Family

The standard Kalman filter requires a model of the given system, and the state of the system is usually described as the following process model:

(1) $$\begin{array}{c} \begin{array}{c} x_{k+1}=A_{k}x_{k}+w_{k} \end{array} \end{array}$$

where $x_{k}$ is the state, $A_{k}$ is the state transition matrix, $w_{k}$ is the state noise with a given covariance of $Q_{k}$, and the measurement model is the following:

(2) $$\begin{array}{c} \begin{array}{c} z_{k}=\;C_{k}x_{k}+v_{k} \end{array} \end{array}$$

where $C_{k}$ is the measurement matrix, $z_{k}$ is the measurement data, and $v_{k}$ is the measurement noise with a given covariance of $R_{k}$. Kalman filtering provides the estimated state $\hat{x}\left(k|k\right)$ by the state’s initialization $\hat{x}\left(0|0\right)$ and covariance P(0|0)=P0:

(3) $$\begin{array}{c} \begin{array}{c} \begin{array}{c} {\hat{x}}_{k|k-1}=A_{k-1}{\hat{x}}_{k-1|k-1}\\ P_{k|k-1}=A_{k-1}P_{k-1|k-1}A_{k-1}^{T}+Q_{k-1} \end{array} \end{array} \end{array}$$

and

(4) $$\begin{array}{c} \begin{array}{c} K_{k}=P_{k|k-1}C_{k}^{T}{\left[C_{k}P_{k|k-1}C_{k}^{T}+R_{k}\right]}^{T}\\ {\hat{x}}_{k|k}={\hat{x}}_{k|k-1}+K_{k}\left[z_{k}-C_{k}{\hat{x}}_{k|k-1}\right]\\ P_{k|k}=\left[I-K_{k}C_{k}\right]P_{k|k-1} \end{array} \end{array}$$

Equations (3) and (4) show that the Kalman filter algorithm has a two-step process. Equation (3) denotes the prediction step, in which the prediction estimates $\hat{x}\left(k|k-1\right)$ are obtained based on the last estimate ${\hat{x}}_{k-1|k-1}$. The second step is the update step shown in Equation (4), in which the estimates $\hat{x}\left(k|k\right)$ are updated using a filter gain $K_{k}$ based on $\hat{x}\left(k|k-1\right)$.

Since most practical systems have nonlinear characteristics, researchers have improved the nonlinear system based on the standard Kalman filter. The nonlinear filtering algorithm, the EKF, uses the Taylor expansion of a nonlinear function to retain the first-order linearization. The study [29] used the EKF to estimate the real-time traffic state in freeway stretches for the nonlinear macroscopic traffic flow model. Next, we provide the system model and then illustrate the EKF estimation method.

The nonlinear relation of the system is described by the following general model:

(5) $$\begin{array}{c} \begin{array}{c} \begin{array}{c} x_{k+1}=f\left(x_{k},w_{k}\right)\;\\ z_{k}=h\left(x_{k},v_{k}\right)\; \end{array} \end{array} \end{array}$$

where f(·) and h(·) are the nonlinear process and measurement equations, respectively. We cannot find the process matrix $A_{k}$ and measurement matrix $C_{k}\;$ in Equations (3) and (4), which are replaced by f(·) and h(·). Therefore, the Kalman filter in Equations (3) and (4) cannot be used to estimate the nonlinear systems in Equation (5).

Then, the EKF obtains the prediction ${\hat{x}}_{k|k-1}$ from the nonlinear transfer $f({\hat{x}}_{k-1|k-1})$. In addition, the innovation $z_{k}-C_{k}{\hat{x}}_{k|k-1}$ can be changed to $z_{k}-h\left({\hat{x}}_{k|k-1}\right)$. The update of the state then becomes

(6) $$\begin{array}{c} \begin{array}{c} {\hat{x}}_{k|k}={\hat{x}}_{k|k-1}+K_{k}(z_{k}-h\left({\hat{x}}_{k|k-1}\right) \end{array} \end{array}$$

We note that the other equations in the standard Kalman filter, such as Equation (4), must use the matrices $A_{k}$ and $C_{k}$. The EKF method obtains these matrices as the following first-order Taylor series expansions: The first-order Taylor expansion of the nonlinear process equation $f(x_{k},w_{k})$ as $\frac{\partial f}{\partial x}{|}_{{\hat{x}}_{k-1|k-1}}=F_{k}$ and $\frac{\partial f}{\partial w}{|}_{{\hat{x}}_{k-1|k-1}}=L_{k}$ and the Taylor expansion of the nonlinear measurement equation $h(x_{k},w_{k})$ as $\frac{\partial h}{\partial x}{|}_{x={\hat{x}}_{k|k-1}}=H_{k}$ and $\frac{\partial h}{\partial v}{|}_{x={\hat{x}}_{k|k-1}}=M_{k}$, where $F_{k}$, $L_{k}$, $H_{k}$, and $M_{k}$ are the transformations from the Taylor expansion based on the nonlinear process equation $f(x_{k},w_{k})$ and the measurement equation $h(x_{k},w_{k})$. Then, similarly to the standard Kalman filter in Equation (4), the estimation covariance and the filter gain are obtained by $F_{k}$, $L_{k}$, $H_{k}$, and $M_{k}$ for the nonlinear system models in Equation (5).

Because the Taylor expansion only has the first order, an EKF is commonly applied to a nonlinear system’s weak case. If the system’s nonlinearity is strong, the error between the approximate linear system of the Taylor expansion and the original nonlinear system leads to a decrease in estimation performance and even to the phenomenon of filter divergence. To maintain the estimation performance, Yang et al. [30] provided the improved federated EKF for multisensor-integrated navigation according to the near-ground short distance navigation applications of small unmanned aerial vehicles (UAVs). The study [31] employed the EKF to fuse the data from multiple heterogeneous sensors, including an inertial measurement unit (IMU), a magnetometer, a barometer, a GNSS receiver, an optical flow sensor (OFS), light detection and ranging (LiDAR), and an RGB-D camera for an unmanned aerial vehicle.

Unlike the EKF, the UKF does not use linearization. Instead, several effective adoption points are used to simulate the state transfer relationship of the nonlinear model. By effectively selecting sampling points, the mean and covariance of the state can be better captured.

In 2002, Julier proposed the UKF method and selected sampling points as the unscented transformation [32]. Later, researchers proposed other unscented transformations such as spherical unscented transformation. The difference from the standard Kalman filter is that the UKF is not just a calculation of the mean and covariance of the state estimation but also the calculation of each sigma point generated by the unscented transformation. Therefore, in addition to the standard prediction and update process, the filter includes generating sigma points and calculating the mean and variance of the current step based on sigma points [33]:

Assuming that the state estimate and its covariance for the previous step have been obtained, namely, ${\hat{x}}_{k-1|k-1}$ and $P_{k-1|k-1}$, the process of unscented transformation is calculated as follows:

(7) $$\begin{array}{c} \begin{array}{c} \begin{array}{c} {\hat{x}}_{k-1|k-1}^{\left(i\right)}={\hat{x}}_{k-1|k-1}+{\tilde{x}}^{\left(i\right)} \end{array} \end{array} \end{array}$$

where

(8) $$\begin{array}{c} \begin{array}{c} \begin{array}{c} {\tilde{x}}^{\left(i\right)}=0,\;i=0\\ {\tilde{x}}^{\left(i\right)}={\left(\sqrt{\left(n+k\right)P_{k-1|k-1}}\right)}_{i}^{T},\;i=1,\cdots ,n\\ {\tilde{x}}^{\left(i\right)}=-{\left(\sqrt{\left(n+k\right)P_{k-1|k-1}}\right)}_{i}^{T},\;i=n+1,\cdots ,2n \end{array} \end{array} \end{array}$$

Then, each sigma point ${\hat{x}}_{k-1|k-1}^{\left(i\right)}$ is predicted and is weighted as

(9) $$\begin{array}{c} \begin{array}{c} {\hat{x}}_{k|k-1}=\sum_{i=0}^{2n}w^{\left(i\right)}{\hat{x}}_{k|k}^{\left(i\right)} \end{array} \end{array}$$

with $w^{\left(0\right)}=\frac{a}{n+a}$, $w^{\left(i\right)}=\frac{1}{2(n+a)},\;i=1,2\cdots 2n$, and a being a positive constant. Similarly, the forward prediction of the measurement and the estimated weight also requires calculating sigma points and the weighted summation. The details of the calculation process can be found in Jin et al. [28]. In our former research [34], we proposed two nonlinear estimation methods based on the EKF and UKF, respectively, for real-time indoor RFID (Radio-frequency identification) tracking. The results show that compared to the EKF, the UKF method can obtain a lower covariance, while the EKF can cost less in calculation. The study [35] developed an adaptive unscented Kalman filter (AUKF) as a vehicle driving state observer based on the seven-degree-of-freedom vehicle model and the Dugoff tire model.

The CKF also uses several points to expand the previous step’s estimation and variance, which is just a special case of the UKF with 2*n* sigma points. Differently from the sigma point of the UKF in Equation (8), ${\tilde{x}}^{\left(i\right)}$ is obtained by

(10) $$\begin{array}{c} \begin{array}{c} \begin{array}{c} {\tilde{x}}^{\left(i\right)}={\left(\sqrt{P_{k-1|k-1}}\right)}_{i}^{T},\;i=1,\cdots ,n\\ {\tilde{x}}^{\left(i\right)}=-{\left(\sqrt{P_{k|k-1}}\right)}_{i}^{T},\;i=n+1,\cdots ,2n \end{array} \end{array} \end{array}$$

with $w^{\left(i\right)}=\frac{1}{2n}$.

We can find that the CKE and UKF are very similar in the form of the algorithm. The CKF is simply a special case of the UKF with 2n sigma points (with no center sigma point). To obtain that, though a different path is followed, in the end, we have a similar algorithm. Therefore, the CUK and UKF are also relatively similar in estimation performance. As an application for the CKF, [36] proposed a strong tracking mixed-degree cubature Kalman filter (STMCKF) method according to the system characteristics of the quadruped robot to obtain the fusion estimation of forward kinematics and the IMU track.

Finally, we would like to discuss the performance of each one of these Kalman filters. The classic Kalman filter requires the system to be linear with Gaussian white noise. It is obviously difficult to meet these requirements in an actual system. Therefore, in practical applications, the classic Kalman filter’s performance cannot reach the optimal estimation performance. However, the classic Kalman filter plays a pioneering role in research, so it is the theoretical basis of other filters. If the system is nonlinear and contains Gaussian white noise, the performance ofthe EKF, UKF, and CKF is different. Most research results indicate that the UKF and CKF are better than the EKF [34]. Meanwhile, the UKF and CKF exhibit almost the same performance. Recently, the Kalman filter has been developed in practical systems, such as the distributed Kalman filter [37] for sensor networks, hybrid Kalman filter [38], and adaptive ensemble square root Kalman filter [39].

### 2.2. Gaussian Mixture Filter and Random Sampling Filter

The Kalman filter given in Section 2.1 is based on single Gaussian noise. However, in existing systems, the distribution characteristics of noise are often more complicated. Suppose the system’s noise can be decomposed into the sum of multiple Gaussian noises, which is what we call a multimode problem. In that case, we can decompose the noise first and then use the filters described in the previous section for each Gaussian noise component.

Suppose p(x) is a mixed Gaussian distribution with *n* Gaussian components

(11) $$\begin{array}{c} p\left(x\right)=\sum_{i=1}^{n}\omega_{i}\;N\left(x;\;\mu_{i},P_{i}\right) \end{array}$$

where $N(x;\;\mu_{i},P_{i})$ is each Gaussian component and the weight satisfies $\sum_{i=1}^{n}\omega_{i}=1$.

**Figure 1:**
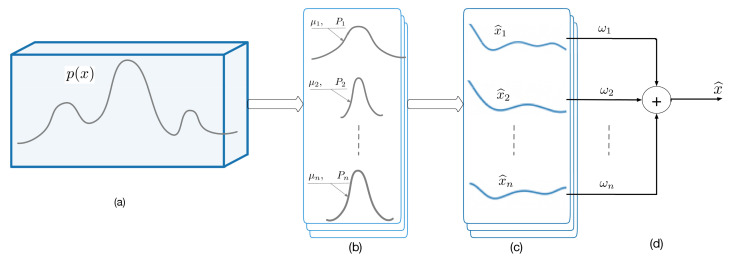
A flowchart of the Gaussian mixture filter. (**a**) The system noise is a complicated one with multiple Gaussian components. (**b**) The complex Gaussian noise of the system is decomposed into several standard Gaussian noises $P_{i}$, i=1,2,......n. (**c**) The state ${\hat{x}}_{i}$ is estimated based on each Gaussian noise. (**d**) The state estimation result based on the complex mixed high noise is obtained by using the estimated result and the weight.

Next, we need to determine the weight $\omega_{i}$, the mean $\mu_{i}$, and variance $P_{i}$ of each Gaussian component. Then, the estimation methods in Section 2.1 can be used to obtain the estimations based on each component with the Gaussian noise. At last, all the estimations can be weight-summed by $\omega_{i}$. A flowchart of the Gaussian mixture filter is shown in Figure 1.

To avoid too many components making subsequent calculations too complicated, it is usually necessary to reduce the number of Gaussian components. In other words, it is necessary to describe as accurately as possible with as few components as possible. The methods of component reduction include retaining those significant components that affect the distribution function by selecting some of the components with larger weights and discarding those that are not significant. Furthermore, by combining similar components, the total number of components is reduced.

Suppose the system’s noise is more complex and cannot be expressed as the approximate sum of multiple Gaussian noises. The particle filters appear for such a problem [40,41]. In that case, a numerical approximation method is required, that is, Monte Carlo simulation. The probability distribution of the state is calculated by selecting multiple samples and using statistical methods. However, the computational overhead is very high. Additionally, in the process of calculation, due to particle degradation, sequential importance resampling processing is required.

**Figure 2:**
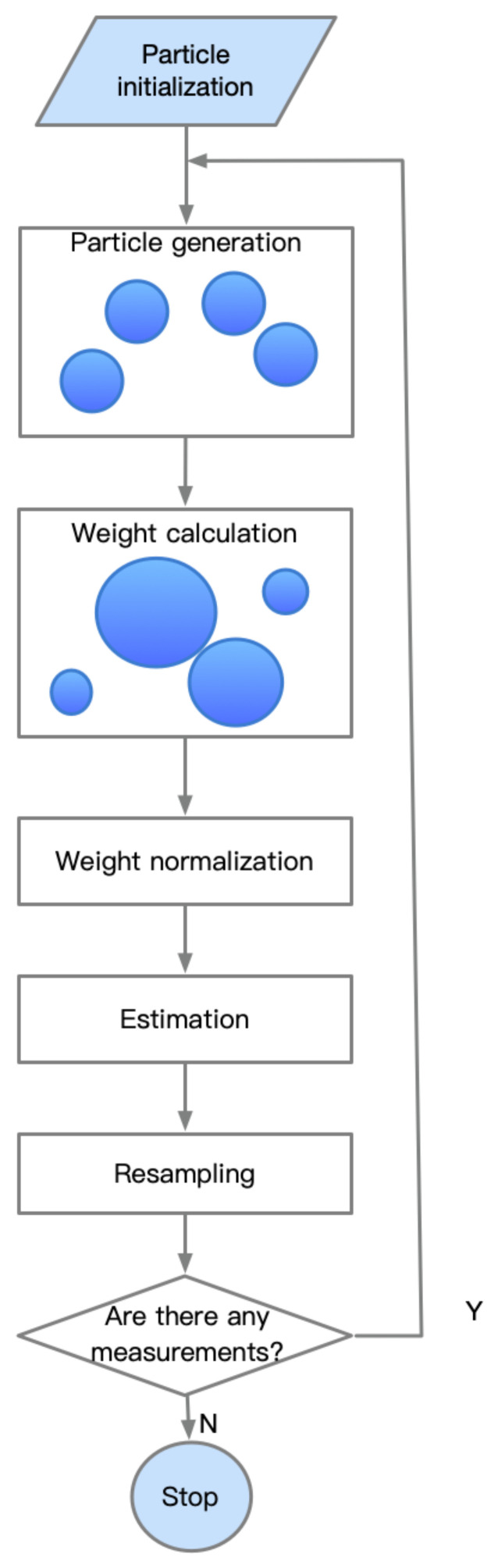
The flow of the particle filter algorithm. The particles are generated firstly, and then, the weights need to be calculated according to the states’ accuracy. Next, the weights are normalized, the state is estimated, and the particles are resampled. The loop is continued until all the measurement data are used.

Figure 2 shows the flow of the particle filter algorithm. A set of particles represents the samples in the distribution. Each particle is assigned a likelihood weight, representing the probability of sampling. The weights tending to be the same is a common problem with these filtering algorithms. However, this can be alleviated by resampling before the weights become too uneven. In the resampling step, particles with smaller weights will be replaced by new particles.

Two reviews provide details about particle filters, [42,43]. The first one focuses on particle filters based on the optimal and suboptimal Bayesian algorithms for nonlinear and non-Gaussian tracking problems. Within the general framework of the sequential importance sampling (SIS) algorithm, several variants of the particle filter are introduced, such as sampling importance resampling (SIR), auxiliary sampling importance resampling (ASIR), and regularized particle filter (RPF). The second one discusses new developments and demonstrates that particle filters are useful in even the largest dimensional geophysical data assimilation problems.

At the end of this subsection, we want to discuss an interesting fact about particle filters and Gaussian mixture filters. That is, particle filters were popular about 20 years ago [44], while Gaussian mixture filters have only become a hot topic in the past decade [45]. The reason is that when the system encounters complex noise, people first use particle filters to complete the task. However, the computational complexity of the particle filter is too large. To improve real-time performance, many research results show a compromise between real-time performance and accuracy. On the other hand, people find that it is possible to use Gaussian mixture filters to complete the task by decomposing complex noise, improving state estimation’s real-time performance while ensuring estimation performance. However, when encountering difficult and incredibly complex noise, particle filters can show better advantages. Therefore, there are still many application examples for particle filters in video tracking [46,47]. However, in target tracking, the Gaussian mixture filter has attracted more attention from researchers due to its small amount of calculation [48,49].

### 2.3. Discussion

Table 1 provides some performance comparison of the filters. We can find that the Kalman filter requires the system to be linear, with Gaussian white noise, and has a low calculation cost. Meanwhile, in practice, it is hard to meet such requirements for the system; therefore, in an actual system, the obtained estimation accuracy is low.

When the system is nonlinear and contains Gaussian noise, the UKF and CKF are the first choices. This is because they do not require a large amount of calculation and can obtain more accurate estimation results than the EKF. Of course, if the system’s nonlinearity is not strong, the EKF is also a good choice. However, if the system contains complex noise, the Gaussian mixture filter is the first consideration. As mentioned earlier, we need to decompose the complex noise into Gaussian noise series and then use the Gaussian mixture filter to complete the estimation task. If the decomposition cannot be achieved effectively, we suggest selecting the particle filter to ensure the actual estimation performance.

To obtain an accurate system model, a system identification method is used to establish the mathematical models of dynamical systems [50,51,52,53], and some identification approaches can be used to establish the prediction models and soft sensor models [54,55,56,57]. Nevertheless, it is still difficult to obtain an accurate model in the existing system. Estimation methods for inaccurate models are also attracting attention.

## 3. State Estimation Based on a Blurry Model

The estimation method in Section 2.1 requires a given system model, including system parameters and the probability distribution of the noise contained in the system. However, the system model parameters and noise distribution characteristics are usually not accurately obtained due to the large scale of the existing system, the complicated relationship between variables, and the influence of the state process, measurement method, surrounding environment, etc.

The estimation performance degradation caused by the low degree of model matching has always existed in actual system applications. It makes the estimation results unable to reach the ideal performance of theoretical research. Simultaneously, it has also promoted the development of classical estimation theory in the past few decades. Below, we introduce the estimation theory and method based on a blurry model from two aspects of a “robust filter” and “closed-loop filter”.

### 3.1. Robust Filter

When the system model parameters are inaccurate, the state can be corrected by modeling the uncertainty term. Based on this, a robust filtering method has been designed to improve the estimation performance. This method first describes the incorrect system model parameters as the perturbation range and then designs the filter gain based on it. The estimated covariance is bounded within the perturbation range of the parameters. The specific methods include guaranteed performance estimation, $H_{\infty }$ filtering, etc. [11,13,58].

To guarantee performance estimation based on the system’s maximum uncertainty, the estimator is designed to minimize the steady-state filter covariance’s upper bound. For example, under the Bayesian framework, Dehghannasiri et al. [59] proposed a robust Kalman filter based on the orthogonal theory, Nishanthi et al. [60] studied the guaranteed performance filtering method for uncertain discrete systems under random hysteresis conditions, and Roy et al. [61] designed a guaranteed performance filter for quantum phase change.

Wang et al. [62] designed a time-varying estimator to realize distributed state estimation based on a type of sensor network with random model parameters and nonlinearity. Under the premise of known topology information, based on Bernoulli’s random distribution, Li and Chang [63] studied the problem of robust filtering and feedback control with random occurrence characteristics for uncertain systems.

The above research results are mainly based on Lyapunov stability. The basic idea is to transform the robust filtering problem into a feasible solution of the Riccati matrix equation and then provide the conditions and robust filters to ensure robust filtering performance. Although the Riccati equation can achieve the method, some parameters still need to be provided in advance. The choice of parameters affects the performance of the result and affects the solvability of the equation. Due to the lack of suitable parameter optimization methods, these parameters need to be set in the design process, which brings conservativeness to the results. On the other hand, Riccati matrix equations are mostly solved by iterative methods, which cannot guarantee the solution process’ convergence.

In the 1990s, with the introduction of the interior point method for solving convex optimization problems, the linear matrix inequality (LMI) method attracted the academic community’s attention. This method can overcome the shortcomings in solving the Riccati equation, provide a convex constraint condition for the problem to be solved, and then obtain the robust filtering problem’s solution by solving the convex optimization problem [64].

The robust $H_{\infty }$ filtering considers the following uncertain discrete linear time-invariant system:

(12) $$\begin{array}{c} \begin{array}{c} x_{k+1}=\left(A+\Delta A_{k}\right)x_{k}+B\omega_{k}\\ y_{k}=\left(C+\Delta C_{k}\right)x_{k}+Dv_{k} \end{array} \end{array}$$

where $x_{k}$ is the state to be estimated, and $\Delta A_{k}$ and $\Delta C_{k}$ are the perturbation of the parameter, and they can be written as

(13) $$\begin{array}{c} \begin{array}{c} \left[\begin{array}{c} \Delta A_{k}\\ \Delta C_{k} \end{array}\right]=\left[\begin{array}{c} H_{1}\\ H_{2} \end{array}\right]F_{k}E \end{array} \end{array}$$

where $H_{1}$, $H_{2}$, and E are the constant matrix with appropriate dimensions, and $F_{k}$ satisfies the following:

(14) $$\begin{array}{c} \begin{array}{c} F_{k}^{T}F_{k}\leq I \end{array} \end{array}$$

If we have the constant β; symmetric positive definite matrix X, Y; symmetric matrix U; and asymmetric matrix M, S, T based on a given constant γ>0, satisfying the following,

(15) $$\begin{array}{c} \begin{array}{c} \left[\begin{array}{cc} X & I\\ I & Y \end{array}\right]>0\\ \left[\begin{array}{cc} X & M\\ M^{T} & U \end{array}\right]>0\\ \left[\begin{array}{cccccccc} -Y & * & * & * & * & * & * & *\\ -I & -X & * & * & * & * & * & *\\ 0 & 0 & -\gamma^{2}I & * & * & * & * & *\\ A^{T} & A^{T}X^{T}+C^{T}T^{T} & I & -X+\beta E^{T}E & * & * & * & *\\ 0 & S^{T} & -I & -M^{T} & -U & * & * & *\\ B^{T} & B^{T}X^{T} & 0 & 0 & 0 & -I & * & *\\ 0 & D^{T}T^{T} & 0 & 0 & 0 & 0 & -I & *\\ H_{I}^{T} & H_{I}^{T}X^{T}+H_{I}^{T}T^{T} & 0 & 0 & 0 & 0 & 0 & -\beta \end{array}\right]<0 \end{array} \end{array}$$

then we can have the robust filter

(16) $$\begin{array}{c} \begin{array}{c} {\hat{x}}_{k+1}=A_{f}{\hat{x}}_{k}+B_{f}y_{k} \end{array} \end{array}$$

with the parameter

(17) $$\begin{array}{c} \begin{array}{c} A_{f}=M^{-1}S\\ B_{f}=M^{-1}T \end{array} \end{array}$$

satisfying the estimation covariance

(18) $$\begin{array}{c} \begin{array}{c} E\left\{e_{k}e_{k}^{T}\right\}<\left[\begin{array}{c} I\\ -I \end{array}\right]{\left[\begin{array}{cc} X & M\\ M^{T} & U \end{array}\right]}^{-1}\left[\begin{array}{cc} I & -I \end{array}\right] \end{array} \end{array}$$

In summary, based on the system’s uncertainty model, robust filtering can perform state estimation based on minimizing the error covariance or performance indicators for all the possible uncertainties of the actual system. However, for a specific practical application, the state estimation accuracy provided is not high.

Therefore, although robust filtering was once a hot research direction in state estimation at the beginning of this century, it has not been widely used in practice because it cannot meet the existing system requirements for state estimation performance. The reason is that it is still very difficult to accurately model the uncertain part first. If the uncertain part cannot be accurately described, then the robust filtering performance is not high. Recently, some research has tried to develop the performance for practice. For example, Liu et al. [65] combined the unscented Kalman filter with the H${}_{\infty }$ filter for state estimation for nonlinear non-Gaussian systems.

### 3.2. IMM and Closed-Loop Adaptive Filter

Based on the fact that “complex system parameters and structures will vary with different applications”, researchers have provided another solution to improve the estimation performance: improve the model to make it more in line with the actual system.

There are two categories for the related methods:

(1) Multiple system models describe parts of the system and then combine to describe the whole system;
(2) Measurement information is applied to continuously optimize the online system model to make the system model as consistent as possible with the current situation.

The former is represented by the “multimodel method” proposed by Bar-Shalom, which simultaneously uses multiple models for estimation, evaluates the estimation results of different models, and then designs a reasonable weighting strategy to obtain the final estimation result [66].

The interacting multiple model (IMM) includes a series of basic models. Then, the estimated results are generated by weighting each model’s estimation so that the tracking performance is better than that of any single model.

The IMM supposes that the probability switching among models obeys a Markov chain, and assumes the prior model number of the system is *N*, the sets of the models are $M\left(k\right)=\left\{m_{i}\left(k\right)\right\},\;i=1\cdots N$, and the transition probability from model *i* at *k* time switching to model *j* at k+1 time is $PT_{ij}$. Then, the model transition probability matrix is as follows

(19) $$\begin{array}{c} \begin{array}{c} PT=\left[\begin{array}{cccc} PT_{11} & PT_{12} & \cdots & PT_{1N}\\ PT_{21} & PT_{22} & \cdots & PT_{2N}\\ \vdots & \vdots & \vdots & \vdots \\ PT_{N1} & PT_{N2} & \cdots & PT_{NN} \end{array}\right] \end{array} \end{array}$$

where each row denotes the current model of the *k* sampling time; each column denotes the prediction model of the k+1 sampling time, i.e., the next sampling time. Note that PT is a given matrix based on prior knowledge.

Then, the probability prediction of the model *i* to model *j* is calculated:

(20) $$\begin{array}{c} \begin{array}{c} PT_{\mu }\left(k+1|k\right)=\left[\begin{array}{cccc} PT_{11}\mu_{1}\left(k\right) & PT_{12}\mu_{1}\left(k\right) & \cdots & PT_{1N}\mu_{1}\left(k\right)\\ PT_{21}\mu_{2}\left(k\right) & PT_{22}\mu_{2}\left(k\right) & \cdots & PT_{2N}\mu_{2}\left(k\right)\\ \vdots & \vdots & \vdots & \vdots \\ PT_{N1}\mu_{N}\left(k\right) & PT_{N2}\mu_{N}\left(k\right) & \cdots & PT_{NN}\mu_{N}\left(k\right) \end{array}\right] \end{array} \end{array}$$

where ${PT}_{ij}\mu_{i}\left(k\right)$ denotes the prediction joint probability of the model *i* transferred to model *j*. Each element in the matrix is added at each column; then, the prediction probability of model *j* at the k+1 time can be obtained:

(21) $$\begin{array}{c} \begin{array}{c} \mu_{j}\left(k+1|k\right)=\sum_{i=1}^{N}{PT}_{ij}\mu_{i}\left(k\right) \end{array} \end{array}$$

Then, the probability prediction of the model is given as

(22) $$\begin{array}{c} \begin{array}{c} \mu_{j/i}\left(k\right)=\frac{{PT}_{ij}\mu_{i}\left(k\right)}{\mu_{j}\left(k+1|k\right)} \end{array} \end{array}$$

The interactive probability is the probability of the model *i* at the current time *k* related to the model *j* in the future k+1 time.

Based on this, researchers have developed multimodel methods for nonlinear and uncertainty parameters. However, this type of method requires multiple models to be calculated in parallel and weighted according to each model’s matching degree with actual system, so the amount of calculation is relatively large. Worse, in practical applications, if the model group contains models with low matching degrees, it will lead to a decrease in the estimation accuracy, so using too many models will not improve the estimation performance.

Another method is to update model parameters in real-time based on state estimation results, such as the expectation–maximization (EM) method and hybrid grid multimodel [67]. Based on this idea, the applicant has also proposed a closed-loop adaptive model [68,69]. For example, Bai et al. [70] built a framework of dynamic noise based on a gyroscope’s noises, and developed the dynamic Allan variance to build the adaptive Kalman filter.

**Figure 3:**
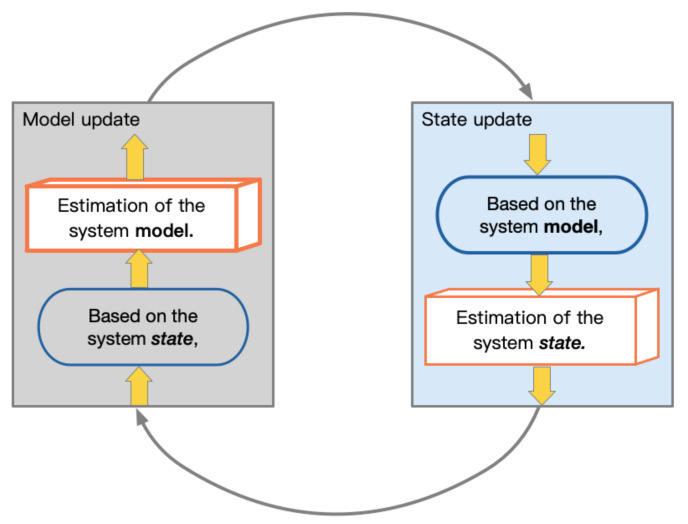
The optimization loop of the system model and state.

This type of model can continuously optimize the system model’s system parameters based on real-time estimation results so that the system model matches the actual system as much as possible. Figure 3 shows the optimization loop of the system model and state. In this type of system, optimal convergence is the most critical issue. We can find that, in such a system, the state and the model are optimized sequentially. The model is initialized first, and then, the state is updated; based on the estimated state obtained, the model parameters are further updated.

However, the optimization based on the current estimation results will cause the model update to lag behind the actual system. The simultaneous update of the state and model parameters may also make the system parameter optimization process unable to converge. These methods can combine identification approaches [71,72,73,74,75] for studying the modeling of dynamic time series and stochastic systems with colored noises [76,77,78,79,80] and can be applied to other fields such as signal modeling, tracking and control systems [81,82,83].

## 4. Data-Driven Modeling by Learning

In this section, we introduce three parts: different deep learning networks, the optimization of their hyperparameters, and one of their learning methods: Bayesian learning.

The network cell is the main part for the data-driven model, which decides the performance of the model. Meanwhile, the choice of hyperparameters has a great influence on the convergence of the network. Therefore, we focus on the hyperparameter optimization in Section 4.2.

As for the measurement for the sensors, the measurement noise has to be firmly considered. Unlike the classic deep learning network, the neural network‘s weight should have the capability to model the input noise characteristics. Section 4.3 describes the so-called Bayesian deep learning network: the methods for Bayesian variational inference and some applications. Table 2 provides the whole picture of the state of the art for the data-driven model method, from which we can find that as the most general application field, “prediction” has the most papers. Meanwhile, recently, some research work has focused on the estimation.

### 4.1. Deep Learning Network

Unlike the traditional models in Section 3, the neural network model based on machine learning does not require other prior physical information or model assumptions [102,103]. In the model training stage, the model is obtained by learning the data’s hidden relationships and knowledge. This type of model can process data with nonlinear characteristics and has been effectively applied to prediction tasks in different fields [104,105].

As the most important machine learning branch, deep learning methods break through the bottleneck of traditional machine learning methods and promote artificial intelligence development. The recurrent neural network (RNN) has become an important process modeling method because it can capture a nonlinear relationship. However, due to the RNN structure’s limitation, the effect will worsen when dealing with longer sequences. The emergence of long short-term memory (LSTM) solved the long-term dependence problem of RNNs [84].

The interior of the LSTM cell has three gating units, which are the input gate, forget gate, and output gate. The calculation process is

(23) $$\begin{array}{c} \begin{array}{c} \begin{array}{cc} f_{t} & =\sigma \left(W_{fx}x_{t}+W_{fh}h_{t-1}+b_{f}\right)\\ i_{t} & =\sigma \left(W_{ix}x_{t}+W_{ih}h_{t-1}+b_{i}\right)\\ {\tilde{c}}_{t} & =\tanh\left(W_{cx}x_{t}+W_{ch}h_{t-1}+b_{c}\right)\\ c_{t} & =f_{t}\cdot c_{t-1}+i_{t}\cdot {\tilde{c}}_{t}\\ o_{t} & =\sigma \left(W_{ox}x_{t}+W_{oh}h_{t-1}+b_{o}\right)\\ h_{t} & =o_{t}\cdot \tanh\left(c_{t}\right) \end{array} \end{array} \end{array}$$

Among them, $o_{t}$ represents the output of LSTM, and $h_{t}$ represents the output of the last layer of LSTM.

Differently from the LSTM, the gated recurrent unit (GRU) network further simplifies the structure of the LSTM network while maintaining the accuracy of the prediction. The GRU model consists of two parts, the update gate and the reset gate.

The formula of the GRU cell is shown below:

(24) $$\begin{array}{c} \begin{array}{c} z_{t}=\sigma \left(x_{t}U^{z}+h_{t-1}W^{z}+b^{z}\right)\\ r_{t}=\sigma \left(x_{t}U^{r}+h_{t-1}W^{r}+b^{r}\right)\\ {\tilde{h}}_{t}=\tanh\left(x_{t}U^{h}+\left(h_{t-1}⊙r_{t}\right)W^{h}+b^{h}\right)\\ h_{t}=\left(1-z_{t}\right)⊙{\tilde{h}}_{t}+z_{t}⊙h_{t-1} \end{array} \end{array}$$

**Figure 4:**
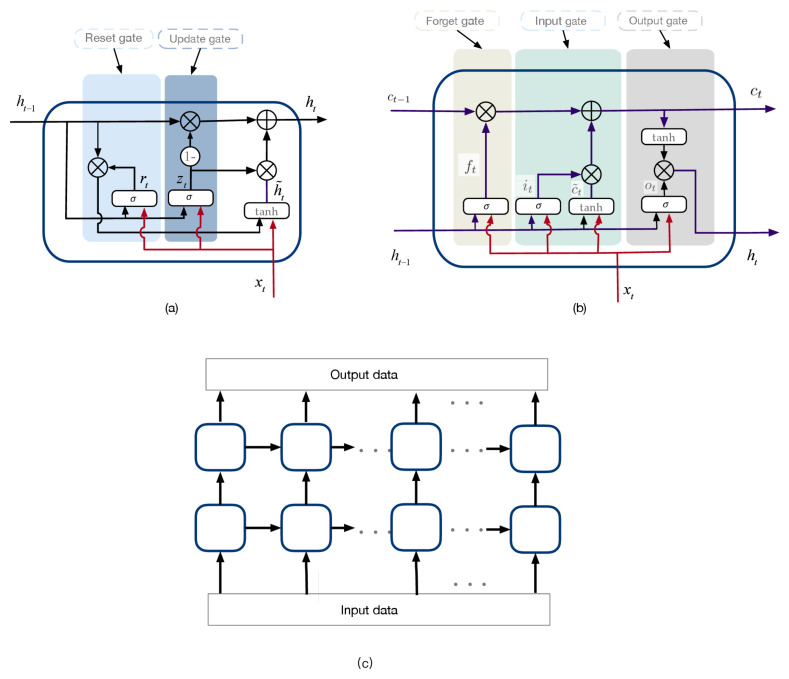
Data-driven model. (**a**) GRU cell; (**b**) LSTM cell; (**c**) deep learning network.

With the GRU and LSTM cell, the network can be constructed. Figure 4 provides an example of a GRU for PM2.5 prediction with two layers [85], in which we find that the deep learning networks still cannot capture the complex dynamic relationship of the data, so the data are decomposed into several components.

In recent years, encoders and decoders based on data modeling have been able to obtain more stable information from data and been suitable for the lengths of input data and output data. One reason is that the encoder extracts features from the input data: the extracted features are the hidden state of the RNN, and the output state of the encoder is used as the initial state of the decoder to obtain the target signal [86]. The other is that the encoder encodes the input data into a context vector and outputs the variable-length sequence as a fixed-length sequence [87].

As the dependency of the input data increases, the performance of data-based modeling declines rapidly. Therefore, by introducing an attention mechanism, different attention weights can be assigned to all the time steps. The relevant encoder hidden state can be adaptively selected in all the time steps, and highly relevant features and output sequences can be extracted. There are two common attention mechanisms: Bahdanau attention [88] and Luong attention [89], also known as additive attention and multiplicative attention.

### 4.2. Hyperparameter Optimization

Due to the increase in data complexity, deep learning methods’ modeling performance needs to be improved. Especially, the hyperparameters of the network greatly influence the modeling performance, so setting the network’s hyperparameters is very important. Hyperparameters determine the model’s performance in deep neural networks; therefore, it is extremely important to optimize the hyperparameters [106].

**Figure 5:**
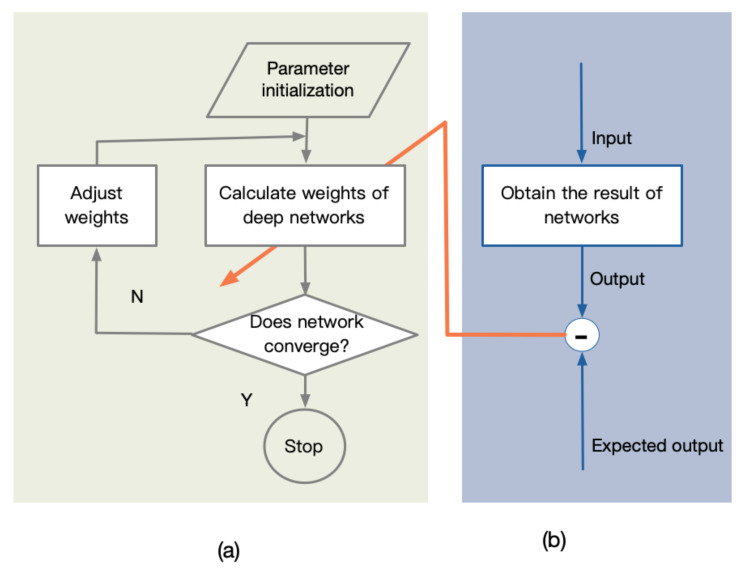
Hyperparameter optimization process. (**a**) The training process; (**b**) The weights’ optimization process.

Hyperparameters need to be set before starting the learning process, not the parameter data obtained through training, but including the number of layers of the network, the number of neurons in each layer, the type of activation function, etc. One hyperparameter, the weight’s training process, is shown in Figure 5, where Figure 5a is the training process for the standard deep learning network to obtain the weights. Based on them, the part shown in Figure 5b evaluates the network performance, defines the weights’ optimization space, and makes it easier to converge to the optimal weights.

At present, hyperparameter optimization methods mainly include manual search, web search, random search, and Bayesian optimization. Among them, a manual search is a process of optimizing hyperparameters manually through trial and error. This method is very time-consuming and relies on experience to determine the optimal hyperparameters. Grid search is traversing all the candidate parameters, trying every possibility, and comparing the values of the objective function one by one to the points that meet the constraints, which is the final result. It is also very time-consuming. Random search is a method of using random numbers to find the optimal solution for function approximation. It is different from the “violent” search method of grid search. This method is based on the theory of probability and continuous randomness within a certain interval. Instead of generating random points with a tendency to calculate the value of the constraint function and the objective function, the values of the objective function are compared one by one for the points that meet the constraint conditions.

Unlike these methods, the Bayesian method will refer to the previous evaluation results when trying the next set of hyperparameters, so it can save a lot of calculations. The Bayesian optimization problem has four parts, which are the objective function, domain space, optimization algorithm, and result history. The principle is to establish a distribution function (probability model) based on the past evaluation results for the objective function, in the constructed domain space, to find the value that minimizes the objective function. Wang et al. [90] used the Bayesian algorithm to optimize the convolution prediction network’s hyperparameters by modeling the complex time-series data sets. The study [91] used a GRU network for each component of power load data, in which Bayesian optimization parameters were used for each subpredictor. The results showed that the Bayesian optimization could effectively improve the prediction accuracy.

### 4.3. The Ability to Model System Noise

The neural network’s ability to model nonlinear relationships has been fully proven theoretically; i.e., the neural network can fit any nonlinear relationship. In practical applications, neural networks, especially deep neural networks, have shown strong advantages in practical application systems, with their powerful nonlinear modeling capabilities. However, an important problem to be faced in the estimation system is the system’s complex noise. Overcoming the influence of complex noise on the estimation performance is one of the important attributes for estimation methods’ performance.

We believe that the way the neural network obtains the weight should consider the input noise characteristics. The Bayesian training process can replace the fixed weights and bias of the traditional neural network. The network training is conducted by sampling, thereby constructing the model’s uncertainty, suppressing the influence of noise, and obtaining more accurate predictions. The neural network output is no longer a fixed sequence but a sequence with a fluctuation range, thereby improving the prediction results’ reliability. Bayesian deep learning networks’ training methods usually include two methods: approximating the integral with Markov chain Monte Carlo (MCMC) and black-box variational inference. We can estimate the true full cost function by Monte Carlo sampling and then backpropagate using the estimated value.

**Figure 6:**
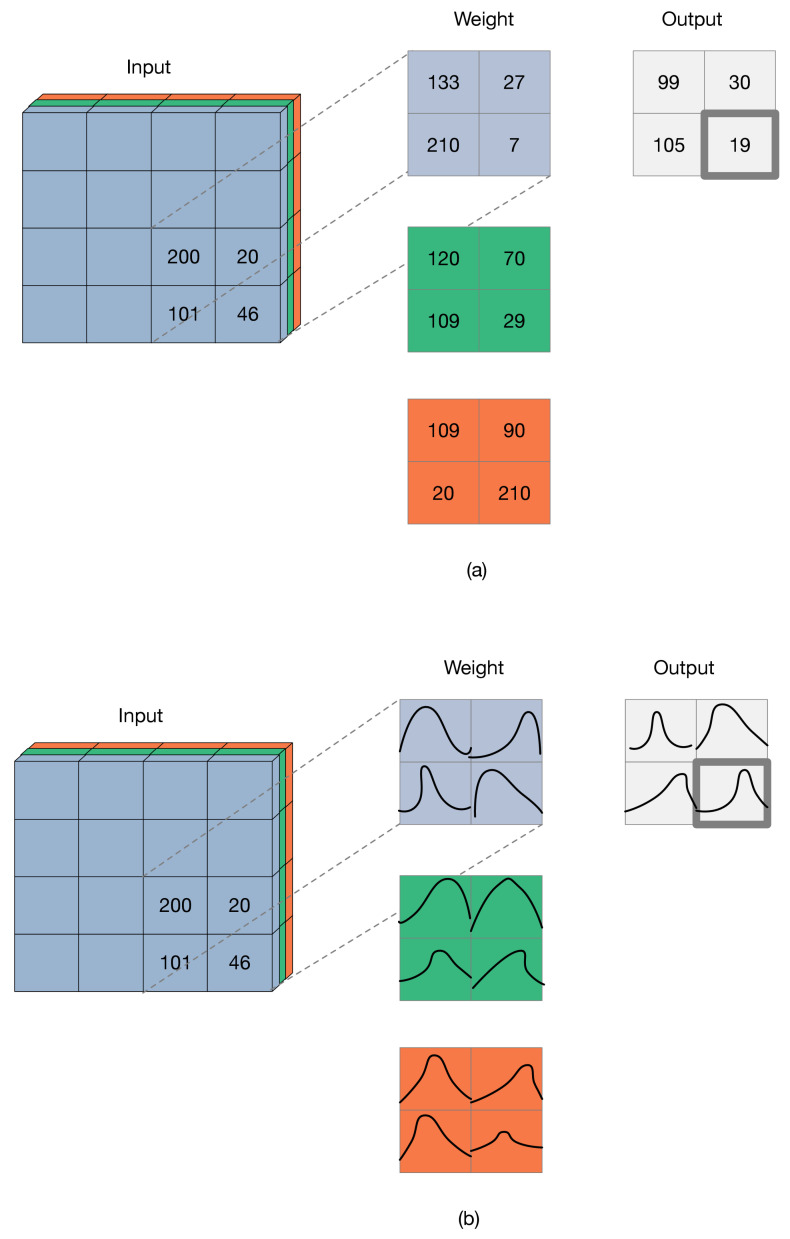
The differences between the Bayesian deep network and non-Bayesian deep network are (**a**) the weight optimization process for the non-Bayesian deep network, where the weight is a certain value, and (**b**) the weight optimization process for the Bayesian deep network, where the weight’s distribution is obtained.

Bayesian deep learning believes that every weight and bias should be a random distribution, not a certain value. Different from the constant weights and biases obtained by non-Bayesian neural network training, the training will obtain the distribution of weights and biases for Bayesian deep learning. The difference between the Bayesian deep network and non-Bayesian deep network is shown in Figure 6.

Given a training data set $D=\left\{\left(x_{1},y_{1}\right),\left(x_{2},y_{2}\right),\ldots ,\left(x_{m},y_{m}\right)\right\}$, suppose we want to obtain the posterior probability p(w∣x,y). Then, according to the Bayes theory, we get

(25) $$\begin{array}{c} \begin{array}{c} p\left(w|x,y\right)=\frac{p(y|x,w)p(w)}{\int p(y|x,w)p(w)dw} \end{array} \end{array}$$

However, the fact is that the denominator has to integrate on the value space of *w*, and the space formed by these weights together will be quite complicated. In general, variational inference is used to solve the integral expression of the denominator. This method uses a simple point distribution q(w) to approximate the posterior probability distribution p(w∣x,y), and uses Kullback–Leibler (KL) divergence to calculate the difference between minimizing the distribution q(w) and p(w∣x,y).

Data-driven methods have been used in state estimation research, which greatly improve the performance of state estimation. The study [107] proposed training a shallow neural network with historical data to “learn to initialize”, that is, to map the available measured values to a point in the neighborhood of the true latent states. The results show that, compared with traditional optimization methods, this machine learning-based optimization method produces superior performance in terms of stability, accuracy, and runtime efficiency. The study [108] was based on the Bayesian inference method for state estimation and used it for sensor transmission power control. Experiments prove that this method can improve the estimation performance.

## 5. State Estimation Based on Hybrid-Driven Methods

With the development of technologies such as sensors, wireless communications, and data storage, more and more historical data are stored in modern complex large systems, forming measurement “big” data. These data contain process information based on the system parameters and noise characteristics that cannot be expressed by the system’s mathematical model. We believe that these data will be used to develop the performance of the simplified system model.

As long as we have enough measurement data, models describing data relationships can be obtained through offline learning without prior knowledge. In recent years, in state estimation, researchers have made some attempts to explore state estimation algorithms that combine the Kalman filter and neural network.

Specifically, they are mainly divided into the following three categories:

Firstly, the network output is the difference between the reference state and the classical estimation, which is used to correct the estimated state based on the system model. For example, Liu et al. [109] trained an LSTM network to predict the difference between the estimated result and the reference trajectory and modify the estimated result. The study [110] proposed a multisensor fusion algorithm for underwater vehicle localization by the augmentation of the radial basis function (RBF) neural network with the EKF, in which the RBF neural network was utilized to compensate for the lack of EKF performance. It simply used the classic state estimation method to evaluate the difference between the actual complex system’s reference trajectory and the estimation result. Similar methods include using learning algorithms to improve the extended Kalman filter [111] and the multimodel extended Kalman filter [112]. In principle, this type of method does not modify the model but uses the difference between the reference state and the classical estimation result to describe the model’s matching degree. When the model error is large, this compensation cannot completely offset the model error.

The second method is to learn the parameters of the system’s model under the framework of Bayesian estimation [113,114]. For example, Zhao et al. [113] trained the network to obtain the state transition matrix, state noise variance, measurement matrix, and measurement noise variance of the system model’s monocular visual tracking. After obtaining these parameters by learning LSTM networks, the state is still estimated based on the system model and the Kalman filter framework. This type of method has the same purpose as the optimization parameter method in Section 3.2. The difference is that the method in Section 3.2 uses a statistical optimization strategy, while here, the learning method is used to obtain system parameters after offline training. However, this method is essentially system parameter identification, which cannot fundamentally solve the estimation performance degradation caused by the system model’s low matching degree, especially the model structure.

The last one is to obtain the estimation state directly [115,116,117,118]. For example, Gao used measurement data as input and the reference state as output data to train the LSTM network [115]; Bai used the NARX (Nonlinear autoregressive with external input) network to learn the complex relationship between the measurement, state, and filter gain in nonlinear systems [116]. This method’s characteristics are as follows: It is necessary to obtain the state reference value through simulation. The system is modeled through supervised learning, and then, the network output is used as the result of the state estimation. This method can use the network model’s ability to model complex data relationships and improve state estimation performance to a certain extent. However, it must be pointed out that the degree of matching between the simulation reference in the method and the actual application system determines the practicability of the model.

## 6. Conclusions

When the model can be accurately obtained or completely consistent with a practical system, the state estimation method using the Kalman filter can produce the best result. For the nonlinear model, the EKF, UKF, and CKF are effective supplements to the standard Kalman filter, and the estimation results for the nonlinear system with Gaussian noise can be obtained. Although this estimation result is no longer optimal, this method is simple and easy to perform effective real-time estimation with, so it has always been used in many applications. However, such methods in existing complex systems, especially in modern complex systems, are not good enough. To reduce the estimation method’s dependence on the model, researchers have provided robust filters, IMM, and closed-loop adaptive filter methods that can use multiple models to describe the system effectively.

Nevertheless, we found that with modern complex systems, the system contains more and more sensors. The system’s internal structure becomes more and more complex, making the modeling of the system more difficult. On the other hand, the system contains more and more sensors, and we can obtain more and more sensor signals. Simultaneously, due to the development of storage technology, we can store these data and generate so-called big data. Based on these big data, we can further describe the model with high accuracy and obtain a more accurate model. Therefore, the state estimation method based on the learning network has been one of the hot spots of people’s attention in recent years.

However, because these methods only use the network to fit the nonlinear system, the network modeling results are not good enough when the system is too complex. Moreover, the traditional neural network modeling ability is further reduced due to sensor noise. Therefore, in recent years, state estimation research has tended to combine the traditional model-based estimation method and the data-driven network model method, in the so-called hybrid-driven state estimation method. In the past three years, related research has shown an increasing trend. It has brought a lot of vitality to the relatively quiet traditional state estimation methods for many years and opened up a new field for studying state estimation methods.

In this review, we summarized and analyzed the three current methods of this research direction. We believe that these studies are very preliminary. There is still a lot of research to be conducted on how to effectively combine the traditional state estimation method based on Bayesian estimation theory with a strict mathematical foundation and the model-driven modeling method that uses the big data complex network model to describe the system. In this regard, we believe that the following research trends are worthy of attention.

The first is how to use sensor-based big data. Compared with the problems studied by traditional deep learning networks, the network model applied to state estimation needs to overcome sensor data noise, which is a more difficult subject for traditional learning networks. Traditional neural network methods can effectively solve nonlinear modeling problems. In theory, researchers have even proved that neural networks can best fit any nonlinearity. Traditional neural networks are prone to overfitting to noise.

Second, the state estimation method should be developed for complex networks. The current hybrid state estimation method does not fundamentally use the advantages of the model-driven and data-driven method. Therefore, the author believes that the research to solve this problem effectively will be of great significance. It can fundamentally combine the advantages of the estimation framework and the nonlinear learning network.

Third, the theory based on the hybrid model needs to be studied in depth. Based on the state of the hybrid drive, the convergence of the estimation method, the requirements for training data, the discussion of real-time performance, etc., are all worthy of our attention.

This review aimed to study the development context of state estimation and describe the development trend of future state estimation. We believe that state estimation will still be an important part of practical systems. For such complex systems, it will bring more difficulties and new solutions to the state estimation methods.

## Figures and Tables

**Table 1:** Comparison of each filter.

Filter	Requirements for the System	Accuracy for a Practical System	Calculation Cost	Description
Kalman filter	Linear, with Gaussian white noise	Low	Low	The requirements for the system are very high, so it is difficult to achieve high accuracy in the actual application system.
EKF	Nonlinear, with Gaussian noise	Medium	Low	The performance of UKF and CKF is better than that of EKF, but their calculation amount is slightly larger than that of EKF.
UKF	Nonlinear, with Gaussian noise	Medium	Medium	
CKF	Nonlinear, with Gaussian noise	Medium	Medium	
Gaussian mixture filters	Nonlinear, with non-Gaussian noise	Medium	Medium	These filters have low requirements for the system. However, the amount of calculation is large.
Particle filters	Nonlinear, with non-Gaussian noise	High	High	

**Table 2:** Data-driven modeling methods with deep learning networks.

References	Network Cell	Hyperparameter Optimization	Type of Network	Purpose
[84]	Long short-term memory (LSTM)	Not mentioned	Classic deep learning network	Classify sequence
[85]	Gated recurrent unit (GRU)	Not mentioned	Classic deep learning network	Forecasting time-series data
[86,87,88]	Recurrent neural network (RNN)	Not mentioned	Classic deep learning network	Machine translation
[89]	Attention-based LSTM	Not mentioned	Classic deep learning network	Machine translation
[90]	Convolution network	Bayesian optimization	Classic deep learning network	Prediction
[91,92,93]	GRU	Bayesian optimization	Classic deep learning network	Prediction
[94]	Bidirectional RNN	Not mentioned	Classic deep learning network	Detection
[95]	ConvLSTM	Not mentioned	Classic deep learning network	Prediction
[96]	RNN	Not mentioned	Classic deep learning network	State estimation
[97]	GRU	Manual search	Classic deep learning network	Prediction
[98]	LSTM	Manual search	Bayesian deep learning network	Prediction
[99]	GRU	Bayesian optimization	Classic deep learning network	Prediction
[100,101]	Multi-layer forward neural network	Not mentioned	Bayesian deep learning network	State estimation

## Data Availability

The data presented in this study are available on request from the corresponding author.
